# Decoding the role of microtubules: a trafficking road for vesicle

**DOI:** 10.7150/thno.110120

**Published:** 2025-04-09

**Authors:** Jiaorong Qu, Jianan Li, Hong Wang, Jianhang Lan, Zixuan Huo, Xiaojiaoyang Li

**Affiliations:** 1 School of Life Sciences, Beijing University of Chinese Medicine, 11 Bei San Huan Dong Lu, Beijing, 100029, China.; 2 School of Chinese Materia Medica, Beijing University of Chinese Medicine, 11 Bei San Huan Dong Lu, Beijing, 100029, China.

**Keywords:** microtubules, vesicle transport, microtubule acetylation, motor protein, cytoskeleton

## Abstract

**Background:** In eukaryotic cells, intracellular and extracellular vesicle transport systems are ubiquitous and tightly linked. This process involves well-defined initiation and termination points, as well as mechanisms for vesicle recycling. During transport, cytoskeletal components serve as "roads" to prevent disordered vesicular movement and to ensure efficient transport, particularly through microtubules. Microtubules primarily facilitate the long-distance transport of vesicles. The dynamic nature of microtubule structure makes its stability sensitive to proteins, drugs, and post-translational modifications such as acetylation, which in turn regulate microtubule-dependent vesicular transport. Furthermore, motor proteins interact with microtubules and bind to cargoes *via* their tail domains, driving vesicle transport along microtubules and determining the directionality of movement.

**Aim of review:** To elucidate the detailed processes and mechanisms of microtubules-regulated long-distance vesicle transport, providing a comprehensive overview of current research in this area.

**Key scientific concepts of review:** This review provides an in-depth analysis of microtubule-mediated vesicle transport, emphasizing the molecular mechanisms involved. It examines vesicle transport between organelles, the impact of microtubule characteristics on this process, and the role of motor proteins in vesicle dynamics. Additionally, it summarizes diseases associated with abnormal microtubule-mediated vesicle transport, aiming to offer insights for the treatment of related conditions.

## Introduction

Cellular cargo transport is an ongoing process that occurs between organelles, from organelles to the plasma membrane, and between cells. Vesicular transport, a primary mechanism of substance movement, facilitates the orderly distribution of cargo within different cellular regions through the formation of transport vesicles 1, 2. Within the cell, membranous organelles, such as the Golgi and the endoplasmic reticulum (ER) can act as sites for vesicle formation, as well as origins or destinations of vesicle trafficking 3, 4. Additionally, vesicular transport plays a crucial role in facilitating material transport between cells 5. During vesicle transport from the origin to the destination, both the direction and speed of vesicles are regulated by the cytoskeletal system. Short-distance vesicle transport, mediated by microfilaments, occurs locally within the cell, while long-distance transport, driven by microtubules, accounts for the majority of the vesicle transport process 6. The α and β-tubulin heterodimers assemble into 13 protofilaments, which laterally bind to form hollow tubular structures cylindrical microtubules with plus ends and minus ends 7. Functionally, microtubules form a dynamic network crucial for structural support, mechanical stability, and signal transmission within cells 8, 9. Notably, the dynamic instability of microtubules significantly influences vesicle trafficking, particularly by serving as tracks for long-distance transport. It has been shown that microtubules are intrinsically dynamic, oscillating stochastically between polymerization and depolymerization in a process called "dynamic instability of microtubules", which underpins their role in vesicle transport 10, 11. More precisely, diverse post-translational modifications of microtubules, such as acetylation, glycosylation, and glutamate residue modification, also regulate the vesicle transport. Notably, the main site of microtubule acetylation, the acetylation of lysine 40 residue (K40) of α-tubulin has garnered significant attention due to its location within the lumen of the microtubule 12, 13. Acetylation of microtubules enhances the stability of their structure and facilitates their self-repair, leading to increased stability of acetylated microtubules 14.

Additionally, vesicles cannot autonomously move along microtubules and require dynein or other motor proteins to act as "feet", facilitating their movement on microtubules. The selection of motor proteins for cargo-laden vesicles and the regulation of the directional movement during transport have emerged as key research topics in vesicle trafficking 15. As mentioned, microtubules have plus and minus ends. Movement toward the microtubules plus end is termed anterograde transport, whereas movement toward the minus end is defined as retrograde transport. The direction of vesicle transport along microtubules is primarily determined by the acting of kinesin and dynein. Specifically, anterograde transport is mediated by kinesin family members, whereas retrograde transport relies on members of dynein family 16, 17. Kinesins and dyneins can collaborate to transport the same cargo, a phenomenon termed bidirectional transport 18. During bidirectional transport, the direction of vesicle movement is ultimately determined by the dominant force in the tug-of-war between kinesin and dynein. In this process, the affinity of cargo to kinesin and dynein, the relative amount of the two proteins, and the loss of kinesin or dynein caused by other reasons, affect the trafficking direction of vesicles.

In addition, abnormalities in microtubule-guided vesicle transport are involved in the pathogenesis of a variety of diseases, especially viral infection 19, 20 and neurological diseases 21, 22. During virus infection and replication, microtubule-guided vesicular trafficking plays a key role in the endocytic virus uptake process and the targeted delivery of genetic material to the host nucleus. Notably, microtubules play a crucial role in nerve cells, particularly in axons and dendrites, where they act as tracks for the transport of vesicles. In this review, we discuss the physiological role of microtubule-mediated vesicle transport both within and between cells. Mechanistically, we explore how microtubule stability and structural dynamics, along with motor proteins and regulatory factors, coordinate vesicle transport.

## Microtubule is involved in the intracellular vesicle transport

### Microtubule-dependent vesicle transport among organelles in cells

Cell homeostasis depends on the ability of distinct cellular organelles to communicate and to exchange cargo. Intracellular protein transport was achieved through multiple pathways, such as selective gating mechanisms, nanotubular structures, and vesicular transport 23. The protein transport among membrane-bound organelles is commonly accomplished through vesicular transport. Notably, the current research indicated that material transport initiated from the ER was not exclusively dependent on vesicles, but rather formed a complex and interconnected tubular network composed of continuous lipid bilayers, known as the ER exit site (ERES). The initial stage of the protein transport involved protein sorting at ERESs and subsequently transition to the Golgi apparatus, which was primarily regulated by two highly conserved protein coat complexes, COPII and COPI. Specifically, COPII localized to the neck region, where it regulated cargo into the ER, while COPI functioned at the distal end, controlling cargo from the ER. In this process, microtubules served as "railways", facilitating the movement of ERES-generated pearl-like membrane vesicles along the microtubules 4. Another study confirmed that vesicular transport between ER and Golgi was closely related to microtubules. Heat shock protein 47 (HSP47) was reported to exit the ER with procollagen and was packaged into transport vesicles destined for the ER-Golgi intermediate compartment (ERGIC) or Golgi apparatus. Meanwhile, the transport vesicles exhibited distinctive directional movement similar to the direction of microtubules and subsequently fused with the cis-Golgi compartment, indicating that microtubules served as the bridges for transporting these vesicles 24. A recent study on the neurotoxin called HIV-1 envelope glycoprotein 120 (gp120) from human immunodeficiency virus-1 (HIV-1) indicated that mannose binding lectin (MBL) facilitated the transport of gp120 from the ER to Golgi vesicles along microtubules. In this specific process, MBL bound to the N-linked mannose residue of gp120, forming an MBL:gp120 vesicle complex. Nocodazole-induced depolymerization of microtubules impeded the transport of the MBL:gp120 vesicle complex, indicating the involvement of microtubules in its transportation 25.

The Golgi apparatus, recognized as the most important organelle for intracellular transport, functions analogously to a "post office" within the cell, dispatching properly modified proteins to various cellular locations in vesicles. While the transport of cargo vesicles containing cell wall components is generally attributed to actin, a noteworthy study has shown that Fragile Fiber1 (FRA1) facilitates the transport of cargo vesicles carrying cell wall components along cortical microtubules, thereby enhancing Golgi vesicle secretion and promoting cell wall formation 26. The biological process of insulin included translation of the insulin precursor in the rough ER and its packaging into secretory vesicles in the Golgi apparatus, which contained insulin polypeptides. It is evident that both microtubules and vesicles play important roles during the biosynthetic process of insulin. A study showed that in β-cells, most microtubules originate from the cytosolic surface of the Golgi membrane. With high glucose stimulation, Golgi-derived microtubules (GDMTs) increased, regulated by cAMP-EPAC2 signaling, suggesting their involvement in the balance between insulin granule secretion and storage 27. Furthermore, as Huntingtin-associated protein 1 (Hap1) was mainly expressed in the brain, recent studies showed that mutant Hap1 protein was associated with microtubules in β-cells, which further destroyed the vesicle transport and reduced the secretion of insulin 28.

Besides, a novel pathway initiating from plasma membrane and transporting extracellular material to mitochondria has been identified, differing from classical endocytosis, which involves vesicle transport from the plasma membrane to early endosomes, late endosomes and lysosomes. In this novel process, FM dye was delivered directly into mitochondria by microtubule-mediated active transport. Specifically, clathrin-conducted endocytosis initiated from the plasma membrane, forming vesicles that were transported to mitochondria through microtubule-mediated active transport 29.

### The mechanism of microtubule-mediated vesicle transport in the apical trafficking

The morphologically polarized cells including highly specialized epithelial cells harbor two types of cell membranes, namely, apical and basolateral membranes. Notably, there was a substantial asymmetry in the number and composition of vesicles produced and released from the apical membrane compared to the basolateral membrane. Microtubule-dependent vesicle transport was reported to be highly involved in the apical trafficking. A novel study investigating the formation of mouse mammary epithelial cells elucidated the role of Huntingtin (HTT) protein in apical polar trafficking and revealed the related mechanism. HTT was found to form a complex with partitioning defective 3-atypical protein (PAR3-aPKC), which was packaged in vesicles and transported along microtubules with the assistant of RAB11A that functioned in apical trafficking of vesicles along microtubules 30. Moreover, another study revealed that the apical vesicles originate from the Golgi apparatus were subsequently transported *via* microtubules to the target membrane, facilitated by the soluble N-ethylmaleimide sensitive factor attachment proteins (SNARE) machinery, which was responsible for membrane fusion. Once treated with colchicine, both the abundance and cellular location of SNARE proteins including VAMP2 and syntaxin were altered 31. These changes were hypothesized to compensate for the absence of microtubule tracks, thereby achieving a comparable level of vesicle membrane trafficking that was disrupted by colchicine. In addition to apical vesicle transport domain, Mx1, a protein belonging to the dynamin superfamily of large guanosine 5′ triphosphatases (GTPases), localized to post-Golgi vesicles. The knockout of Mx1 reduced the dynamics of apical protein secretion and the association of Kif5B with apical transport vesicles 32. Recent studies have shown that the E3 ubiquitin ligase Mindbomb 1 (MIB1) mediates cytoskeletal apical extension and vesicle trafficking during zymogenic cell maturation 33.

## 3. Microtubule is involved in the intercellular vesicle transport

**Figure 1:**
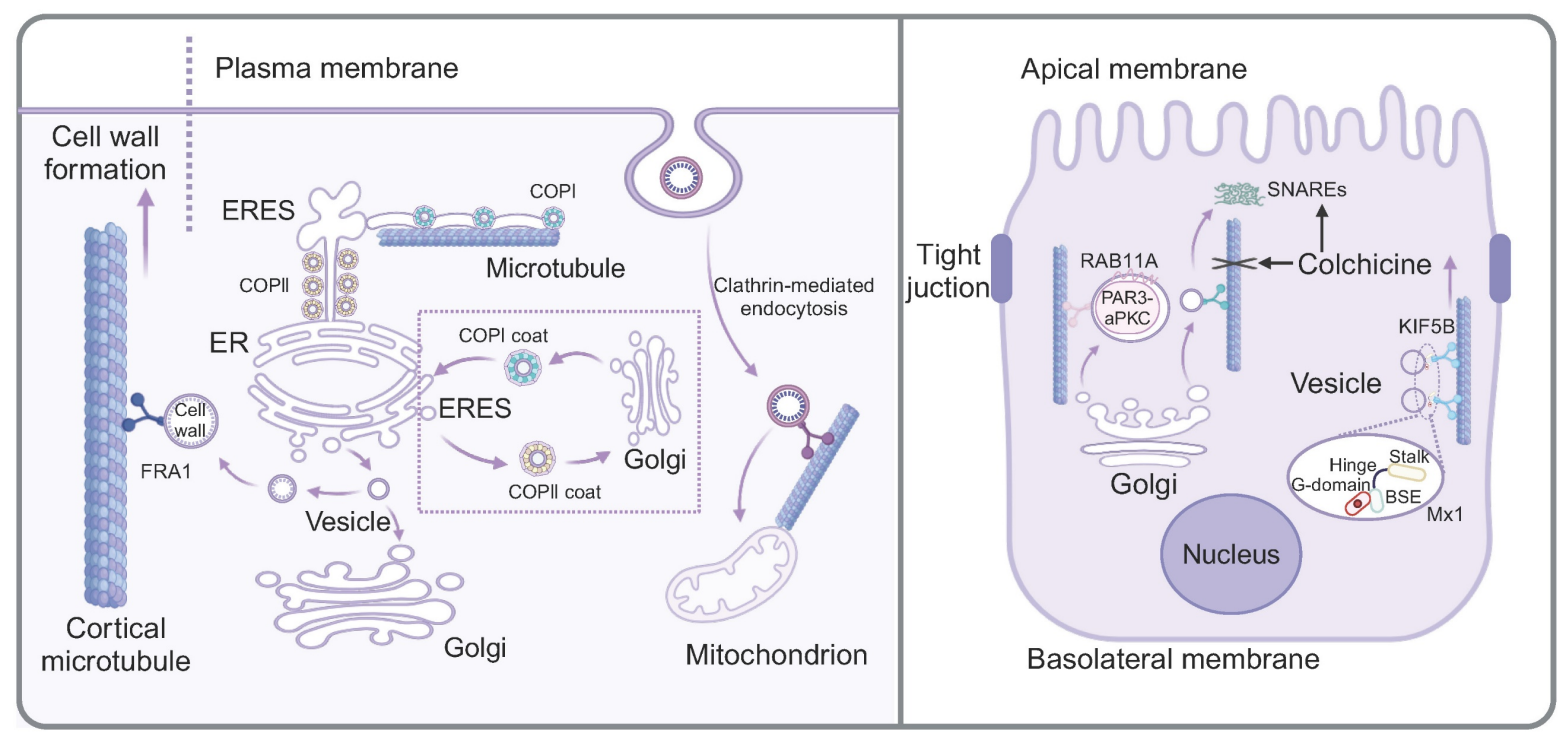
** Microtubule-mediated vesicle transport in the intracellular environment.** The left panel shows that FRA1 facilitates the transport of cargo vesicles containing cell walls components along cortical microtubule. The protein transport between ER and Golgi involves two highly conserved protein coat complexes, COPII and COPI. ERES generate pearl-like membrane vesicles that transport along microtubule. During apical trafficking of vesicles along microtubules (the right panel), RAB11A plays a supportive role. Colchicine not only affect the microtubule stability, but also alter the abundance and cellular location of SNARE proteins. Mx1, localized on post-Golgi vesicles, increase the dynamics of apical protein secretion and the association of Kif5B with apical transport vesicles.

In addition to serving as tracks for intracellular material transport (**Fig. 1**), recent studies have found that microtubules can also form channels between cells, thereby facilitating the transport of vesicles. It has been shown that macrophages could internalize vesicles containing carboxyl-modified quantum dots (cQDs) through a process mediated by microtubules. Specifically, molecular motors driven microtubule cytoskeletons between macrophages, enhancing vesicle transport through this network 34. Membrane nanotubes (NTs), usually contain filamentous actin (F-actin) and microtubules that usually existed in thick NTs, are elongated cellular swelled structures connecting cells. It has been shown that mature B lymphocytes contain microtubules in their thick tunneling nanotubes (TNTs). CD86, a significant stimulus in cellular interactions, facilitates transport between B cells and macrophages. Briefly, in B cells, transport predominantly occurs through nanotube membrane channels, whereas in macrophages, it primarily involved microvesicle-like structures within TNTs 35. The study found that excess Cu^+^ induced the release of ATP7B-containing vesicles from the trans-Golgi network (TGN), which were transported to the basolateral membrane. Then, these vesicles containing ATPase copper transporting beta (ATP7B) were transported from the basolateral to the apical domain through microtubules, which were regulated by Rab11a and subapical compartment. Finally, the vesicles targeted and transported towards the bile duct to metabolize excess Cu^+^ 36. We propose that microtubules mediate intercellular material transport by directly forming channels that guide the random, disordered movement of vesicles destined for secretion into the extracellular space. This mechanism significantly enhances the efficiency of intercellular material transport.

## 4. The influence of microtubule dynamic instability on microtubule-mediated vesicle transport

### The physical features of microtubules determine the vesicle trafficking

Microtubules form a complex physical network structure for vesicle transport. Recent studies have found that the transport behavior of vesicles changes at microtubule junctions, with important implications for understanding complex vesicles transport. Overall, the trajectory of these complex vesicles consists of linear segments and intermittent pauses 37. The presence and size of the microtubule overlaps determined the localization of Sec6, a key component that regulated the cellular secretion pathway by modulating the positioning and fusion of membrane vesicles. In cells lacking the microtubule dynamics regulator Kin4-Ic, the shortening of the overlapping microtubules was delayed at anaphase onset, resulting in the changed location of Sec6 38. Moreover, the length of microtubules was another physical factor determining the direction of vesicle trafficking. In methyl-CpG-binding protein 2 (MECP2)-deficient mice, the growth rate of microtubules and the trafficking of vesicles along microtubules were disturbed, which was reversed with the treatment of epothilone D (EpoD) through enhancing microtubule growth and guiding vesicle transport 39. Furthermore, during long-distance transport along axons, the length of microtubules was critical for vesicle transport, especially at the end of microtubules. If the length of microtubules was limited, vesicle trafficking paused briefly until microtubules elongated 40.

### The acetylation of microtubules controls the vesicle transport

Microtubule acetylation helped the axonal transport of vesicles along microtubules. Recent studies have suggested that vesicles containing a high concentration of the acetyltransferase, alpha-tubulin N-acetyltransferase 1 (ATAT1), played a significant role in the acetylation of microtubules in axons. Specifically, ATAT1 was enriched on the cytoplasmic side of the moving vesicles that transported along the microtubule, which promoted the acetylation of α-tubulin and subsequently accelerated the transport of vesicles along axons 41. Moreover, another research has demonstrated that p27Kip1 stabilizes ATAT1 through its intact C-terminal domain, thereby promoting the acetylation of α-tubulin, which in turn impacts the rate of vesicle transport along microtubules 42. In-depth research showed that microtubule acetylation was involve in the vesicle distribution, orientation, run length and velocity, which were highly associated with the kinesin KIF1C. With the treatment of a microtubule acetylation inhibitor, tubacin, the direction of the KIF1C-positive vesicles shifted from anterograde transport to disorganized transport, showing simultaneous bidirectional movement within the cell. Meanwhile, the velocity and run length of KIF1C-positive vesicles were also altered during their disorganized transport 43. Moreover, microtubule acetylation showed important effects on protein trafficking and was relevant to the transport of vesicles along microtubules. Ethanol-induced hyperacetylation of microtubules restricted transcellular transport, obstructing vesicle transport along microtubules. The specific mechanism underlying this phenomenon might be associated with enhanced binding of dynein to microtubules, resulting in stalled vesicle transport 44.

### Microtubule stability affects the process of vesicle transport

**Figure 2:**
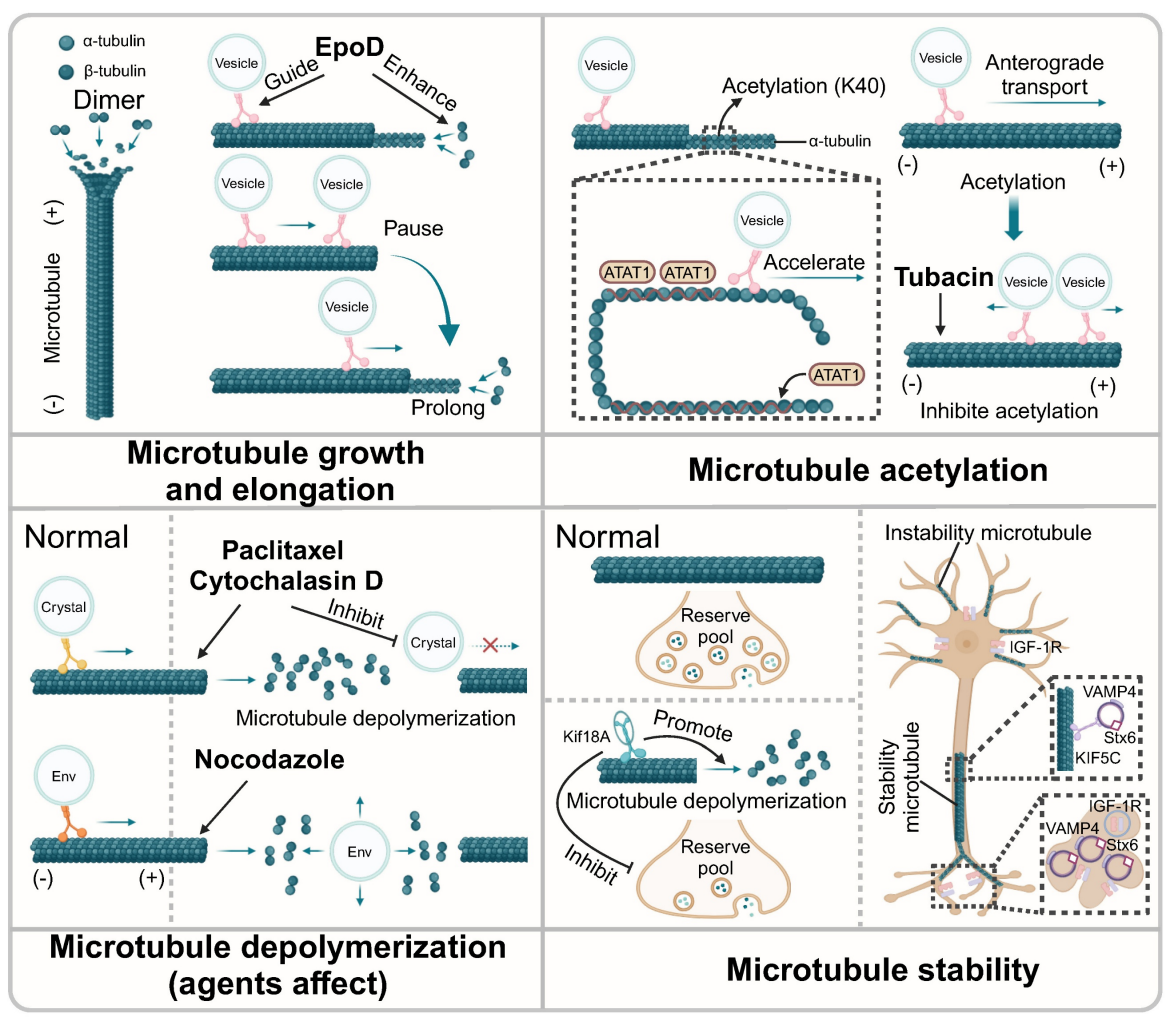
** The influence of microtubule dynamic instability on microtubule-mediated vesicle transport.** EpoD enhances the growth of microtubules and guides the transport of vesicles along microtubules. Vesicle transport will pause briefly at the ends of microtubules until the microtubules are extended. Moreover, the acetylation of microtubule promotes vesicle trafficking. ATAT1 promotes the acetylation of α-tubulin and the transport of vesicles along axonal. Tubacin alters the direction of the vesicles *via* inhibiting microtubule acetylation. Agents that promote microtubule depolymerization inhibit the transport of vesicles along microtubule. Notably, nocodazole could affect the linear movement of vesicle containing Env that mediated by microtubules. Also, Kif18A promotes the depolymerization of microtubule, affecting the refilling of the synaptic vesicle pool. In stable microtubules, KIF5C assists the transport of vesicle along microtubules. SNARE proteins are transported to microtubule-enriched neurites, which subsequently produced vesicles.

As an important component of the cytoskeleton, microtubules maintain dynamic equilibrium through polymerization and depolymerization. Notably, once the balance is disturbed, the unstable microtubules may lose the ability to control the vesicle trafficking. TUBB, as one of the tubulin coding genes, plays an important role in the microtubule dynamics. In TUBB mutant fibroblasts, microtubule polymerization and vesicle transport were disrupted, resulting in the impaired trafficking of EGF and TFs 45. Studies indicated that microtubule depolymerization impacted the filling of the vesicle pool, which comprised the collective population or reservoir of vesicles within a cell. Specifically, Kif18A, a motor protein, could promote the depolymerization of microtubules, thereby inhibiting the refilling of the synaptic vesicle pool 46. In addition, the over-assembly of microtubules also affected the transport of vesicles. As reported, the overexpression of α-synuclein disrupted vesicle transport along microtubules by inhibiting endocytosis of vesicles and continuously stimulating the exocytosis of vesicles, which could be solved by microtubule depolymerizing agents 47. It has been shown that on stable microtubules, kinesin KIF5C preferentially translocates SNARE proteins Stx6 and VAMP4 to stable microtubule-enriched neurites, which are subsequently stimulated by IGF-1 receptors (IGF-1r) to produce vesicles 48. Interestingly, this process was determined by the stability of microtubules rather than microfilaments. Thus, this study suggested microtubules served as organized tracks to guide vesicle trafficking, thereby ensuring the efficient process of material transportation. In addition, many small-molecule agents could interfere with vesicle transport by influencing the polymerization and depolymerization of microtubules. Specifically, with the treatment of paclitaxel, a microtubule inhibitor or cytochalasin D, an F-actin depolymerization agent, the trafficking of vesicles packed crystals along microtubules was suppressed in *Plasmodium* 49. Moreover, another study showed that vesicles carrying envelope glycoprotein (Env) were transported in an anterograde manner within cells. When microtubules were disrupted by the inhibitor nocodazole, Env-containing vesicles lost linear movement, indicating that the transport of Env-containing vesicles is mediated by microtubules 50. Interestingly, another study revealed that diclofenac, a nonsteroidal anti-inflammatory drug (NSAID), had the potential to specifically promote microtubule depolymerization and inhibited autophagy by relying on microtubule-mediated transportation and fusion of autophagy vesicles, which positioned diclofenac as a candidate for the treatment of certain cancers 51. Thus, microtubule features themselves determine vesicle transport efficiency, acting as specialized tracks (**Fig. 2**).

## 5. Microtubules and microfilaments are able to interact with each other in vesicle transport

Recent studies have revealed the function of microtubules and microfilaments in the processes of vesicle secretion, highlighting their contributions to the trafficking of secretory vesicles. The long-distance transport of vesicles occurs along microtubules mainly by kinesin and dynein motors, whereas short-distance transport is conducted on microfilaments through the action of myosin motors 6, 52. Studies showed that F-actin mainly played a role in rapid recruitment of synaptic vesicles. Microfilament depolymerizing agent latrunculin A blocked synaptic vesicle transport, while microtubule depolymerizing agent vinblastine directly disturbed long-distance synaptic vesicle transport, which was independent of F-actin-mediated vesicle transport 53. When microtubule-depolymerizing agents (e.g., nocodazole) or actin-depolymerizing agents (e.g., latrunculin and cytochalasin D) were applied, vesicle transport became dispersed instead of the original transport to the periphery. The dispersed transport of vesicles slowed down the filling of the vesicle pool, which in turn affected vesicle secretion 54. Notably, microfilaments and microtubules complement each other in mediating vesicular transport. In instances of microtubule depolymerization, microfilament-mediated vesicle transport could partially compensate for the loss of microtubule-mediated transport. Specifically, the application of microtubule depolymerization agents including colchicine and nocodazole, led to increased actin filaments polymerization and the formation of actin coat on vesicles 55. Indeed, microfilament depolymerization also interrupted vesicle transport. It was reported that cortical actin depolymerization induced by clostridium difficile transferase (CDT) increased fibronectin secretion and relocated vesicle trafficking, resulting in misdirected vesicle trafficking to apical membrane and membrane protrusion sites 56. Mammalian diaphanous protein 1 (DIAPH1) could regulate actin polymerization and microtubule stabilization to coordinate microtubule-dependent vesicle trafficking, indicating that both microtubules and microfilaments were essential in the vesicle transport 57. In conclusion, microfilaments and microtubules not only serve distinct functions but also cooperate or even complement each other, ensuring orderly cargo transport within cells.

## The key mediators are involved in directional vesicle transport along microtubules

**Figure 3:**
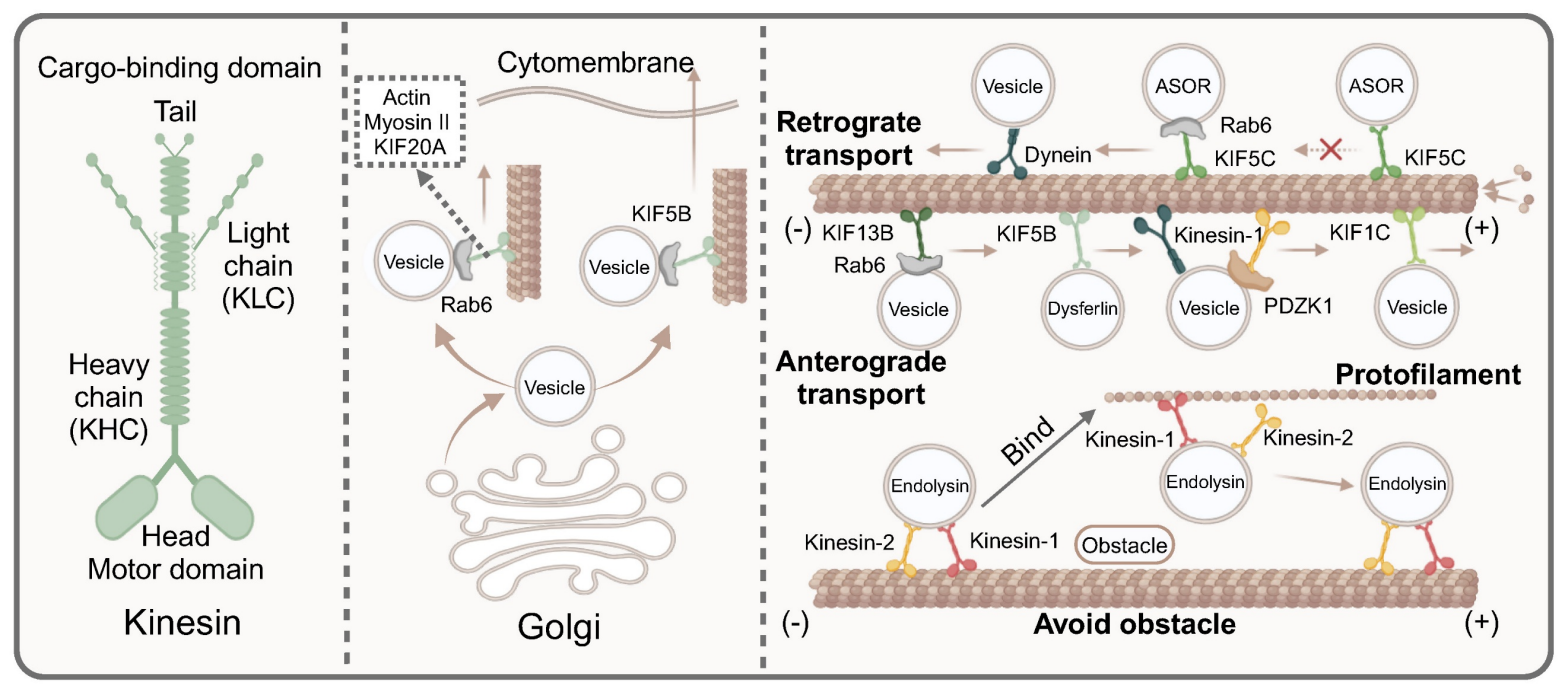
** The key mediators of guiding directional vesicle transport on microtubules.** Most kinesin contains two heavy chains and two light chains, which consist two main domains, namely the motor domain and the cargo-binding domain. Actin myosinII and KIF20A affect the trafficking of Rab6-positive vesicles along microtubules from the exit Golgi/trans-Golgi network membranes. KIF5B promotes the Rab6-positive vesicles from Golgi to the cell periphery. Dyneins facilitate retrograde transport, while kinesins including KIF13B, KIF5B, KIF1C, kinesin-1 mediate anterograde transport. However, some kinesins drive vesicles towards the minus of microtubules, such as KIF5C and KIFC1. Kinesin proteins avoid obstacles in the transport process. When encountering obstacles, kinesin-1 bind to the protofilament to bypass the obstacle.

As summarized, alterations in microtubule dynamics, such as stability, acetylation, depolymerization and polymerization, all may affect the transport of vesicles along microtubules. In addition, kinesin, dynein and a variety of other proteins could guide vesicle transport direction, avoid obstacles, and adjust transport speed, all of which were essential for vesicle transport along microtubules. Importantly, one of the key characteristics of microtubule-mediated vesicle transport is its directionality, which is determined by the polarity of microtubules and the presence of motor proteins. Motor proteins acted like to the "feet" of vesicles, helping them walk on microtubules. Therefore, we would further summarize the specific mechanism of motor proteins (**Fig. 3**).

### Motor proteins compete in vesicle trafficking along microtubules like a tug of war

Since the transport of cargo was bidirectional, the overall net direction was achieved through multiple cycles of bidirectional movement. A complex mutual offset effect took place within the transport, enhancing transport stability and enabling cells to withstand greater disturbances during network transportation 58. Specifically, the direction of vesicle transport along microtubules was influenced by dynamic motor proteins, primarily kinesin and dynein, which respectively dominated different transport directions. Specifically, both anterograde and retrograde transport of vascular endothelial growth factor (VEGF) vesicles along microtubules existed in the cells. The rate of VEGF vesicles in anterograde transport was twice as fast as its retrograde transport, despite their near-comparable speeds. The transport of VEGF vesicles along microtubules was mainly driven by kinesin-1b. Interestingly, kinesin-1b and VEGF vesicles had co-transport in both directions, which indicated that kinesins not only promoted anterograde trafficking of VEGF vesicles, but also played a role in retrograde trafficking 59. Moreover, newly identified proteins were involved in regulating bidirectional vesicle transport. As reported, the presence or absence of PDZK1 directly determined the domination of the kinesin guided positive transport or dynein guided negative transport. Specifically, PDZK1 recruited kinesin-1 to mediate anterograde trafficking of vesicles along microtubules. Nevertheless, in the absence of PDZK1, vesicle trafficking was predominantly driven by dynein, directing movement toward the minus end of microtubules 60. Functionally acquired mec-12/tubulin mutants caused mistargeting of synaptic vesicle and a synapse-swelling phenotype, exacerbated by enhanced interaction with microtubule-associated dynein due to a single amino acid substitution. Consequently, dynein proteins prevailed in the tug-of-war, mislocalizing the vesicles to an incorrect, non-axonal vesicle compartment 61. As research progresses, the kinesin family continues to reveal its complexities. It was well known that the stalk and motor domains of KIF1C affected vesicle trafficking by interacting with microtubules. Furthermore, protein tyrosine phosphatase N21 (PTPN21) could bind to the stalk domain of KIF1C, thereby increasing the landing rate of KIF1C on microtubules. In a word, PTPN21 could trigger the autoinhibition release mechanism, which prevents the tug-of- war scenario during the transport of vesicles carrying cargoes along microtubules and promote the anterograde transport 62.

### Kinesin motors generally mediate vesicle directional transport along microtubules

Kinesins are primarily composed of two heavy chains (KHCs) and two light chains (KLCs). Structurally, kinesins can be divided into two main domains: the motor domain and the cargo-binding domain. The motor-binding domain, located at the amino terminal of KIF5, is responsible for binding to microtubules. In contrast, the cargo-binding domain at the C-terminal domain interacts directly with cargo adaptor proteins or connects to cargo through the indirect action of KLC 63. As reported, KIF5B driven dysferlin-containing vesicle transport along microtubules in both normal and injured myocytes. Notably, in injured myocytes, dysferlin-containing vesicles fused to form large cytoplasmic vesicles, whose transport relied on microtubules and KIF5B 64. Most kinesins exhibited high affinity for microtubule plus-ends, enabling anterograde vesicle transport. It was reported that kinesin motors driven the transport of RAB GTPases Rab6-positive vesicles from the Golgi to the cell periphery. Specifically, KIF5B played a dominant role in vesicle secretion, while KIF13B promoted the anterograde transport of Rab6 vesicles to the ends of newly growing microtubules 16. Furthermore, actin, myosin II and KIF20A also participated in the trafficking of Rab6-positive vesicles by directly interacting with Rab6, driving vesicle trafficking from the exit Golgi/ trans-Golgi network (TGN) membranes along microtubules 65. However, some kinesins could drive vesicles towards the minus end of microtubules. KIFC1, a C-type kinesin in the kinesin-14 family, was a minus-end-directed motor protein critical for microtubule crosslinking and spindle assembly 66. Another study suggested that KIF5C was also involved in transporting vesicles towards the minus-end of microtubules. Typically, cargo was transported by early endocytic vesicles, which then fused and sorted the cargo, split to form vesicles carrying different functions to the receptor. However, Rab1a knockdown resulted in defective processing of asialoorosomucoid (ASOR) protein, leading to abnormal transport characterized by a slower degradation rate of ASOR. Specifically, after Rab1a knockout, ASOR protein failed to dissociate from its receptor and could not arrive at lysosomes for degradation, indicating a defect in early endosome sorting, which led to abnormal transport. The underlying issue was the absence of the negative terminals of the vesicle protein Kif5C, which impaired the transport capacity of vesicles along microtubules in the absence of Rab1a 67. In addition, kinesins not only played a directional role in the transport of vesicles along microtubules, but they also actively and effectively navigated obstacles encountered during this process, thereby enhancing vesicular transport efficiency. Kinesin-1 and kinesin-2 assisted in transporting endolysin vesicles along microtubules. When faced with obstacles during transport, kinesin-1 could bind to the protofilament, allowing it to circumvent the obstruction 68.

Neurons communicate through the regulated fusion of neurotransmitter-containing vesicles, a process facilitated by microtubule-controlled directional vesicle transport. For instance, the transport of dense core vesicles (DCVs) that sprout from the Golgi network and transported to the axon were mediated by microtubules 69. Kinesin motors exhibited varying affinities for microtubules in different neurites at distinct stages of neuronal development. This variability subsequently resulted in the selective transport of vesicles along microtubules 70. KIF1/kinesin-3 could regulate the selective trafficking of vesicles along microtubules. Specifically, three kinds of vesicles containing lysosomal-associated membrane protein 1 (LAMP1), synaptic, and brain-derived neurotrophic factor (BDNF) respectively were transported among the different branches in neurons. This observation indicated that the axonal transport of vesicles along microtubules was not a random process, but rather a selective one, which was closely related to growth cone (GC) motility and branch length regulated by KIF1/kinesin-3 71. Notably, in neurons, microtubules were uniformly organized with their plus (+) ends oriented towards the axonal terminals and their minus (-) ends towards the cell body. Kinesin motors primarily moved towards the plus end of microtubules, which was called anterograde axonal transport. As reported, propofol could inhibit kinesin motors KIF5C and KIF1A, resulting in the reduction of vesicle transport rate, microtubule-mediated vesicle fusion and the inhibition on anterograde axonal transport 72. Indeed, apart from axons, microtubule-dependent vesicle transport also taken place in dendrites, relying on the assistance of kinesin proteins. Furthermore, microtubules polymerizing into dendritic spines served as a direct pathway for kinesin-driven transport of specific cargo, including synaptotagmin-IV (syt-IV)-containing vesicles, into spines undergoing activity-dependent plasticity. When kinesin KIF1A was knock down, syt-IV-containing vesicles frequently fused and diffused into the dendritic spines, disrupting syt-IV trafficking 73.

### The important role of other proteins in directional transport of vesicles along microtubules

However, except for kinesin and dynein, there are other proteins that played integral roles in directional transport of vesicles along microtubule. Recently, p0071 was found to interact with the kinesin proteins including KIF3B and the kinesin cargo adaptor protein 3 (KAP3) to participate in the anterograde transport of vesicles containing chromogranin A (CgA). In the absence of p0071, the motor proteins were more readily dissociated from the vesicle, impairing their ability to move along microtubules 74. Furthermore, syntabulin played an important role in transporting PICK1 (protein interacting with C-kinase 1)-containing vesicles along microtubules. Syntabulin assisted in the formation of microtubule transport structures by recruiting PICK1 to facilitate PICK1-containing vesicle transport. Meanwhile, syntabulin regulated vesicles axonal targeting and trafficking 75. In addition, PDZ domain containing 1 (PDZK1) recruited molecular motors to transport rat OATP1A1 (rOATP1A1)-containing vesicles along microtubules to the plasma membrane, thus promoting the uptake and metabolism of substances in liver. Interestingly, a study revealed that most endocytic vesicles containing rOATP1A1 also contained rOATP1A4. However, the transport of rOATP1A4-containing vesicles was found to be independent of PDZK1, indicating that rOATP1A4 might exhibit hitchhiking behavior 76.

## Mechanisms that regulate the speed of vesicle transport along microtubules

It is intriguing that the microtubule depolymerization, often likened to the disassembly of a 'railway', results in an increase in vesicle transport speed. However, this increase in speed is accompanied by more erratic direction, highlighting the significance of cellular cytoskeleton remodeling. This process, primarily involving the restructuring of microtubules, played a pivotal role in regulating both the speed and direction of vesicle trafficking 77. Additionally, some proteins and chemical agents were specifically involved in modulating the speed of vesicle transport. A giant Drosophila Ankyrin2 isoform Ank2-XL and microtubule-associated protein Futsch were reported to be involved in maintaining the proper organization and arrangement of microtubules in axons. Once these proteins were mutated, the spacing between microtubules was substantially reduced, resulting in a decrease in the rate of anterograde axonal transport for synaptic vesicles 78. Moreover, RacC played an important role in the trafficking of ACA-YFP vesicles along microtubules. In racC (-) cells, the trafficking of vesicles became disoriented and slow 79. The trafficking of vesicles along microtubules was typically driven by proteins, especially motor proteins. However, an intriguing study has demonstrated that ATP can also drive the transport of brain vesicles. Studies revealed that numerous glycolytic enzymes were associated with vesicles. When these vesicles were co-incubated with GAPDH substrates, they were capable of producing ATP. This increase in ATP production subsequently enhanced fast axonal transport, which was mediated by ATP kinase and dynein 80. Moreover, a synthetic corticosteroid medication, dexamethasone (DEX) enhanced the movement of quiescent vesicles located outside synapses and increase the speed of microtubule-based vesicle transport. Mechanistically, DEX upregulate the expression of huntingtin protein, a positive regulator of microtubule-dependent vesicle transport in neurons, thereby promoting the trafficking speed of vesicles along microtubules 81.

## Microtubule-guided vesicular transport participants in diseases processes

### Microtubule-dependent vesicular transport in viral infection and replication

**Figure 4:**
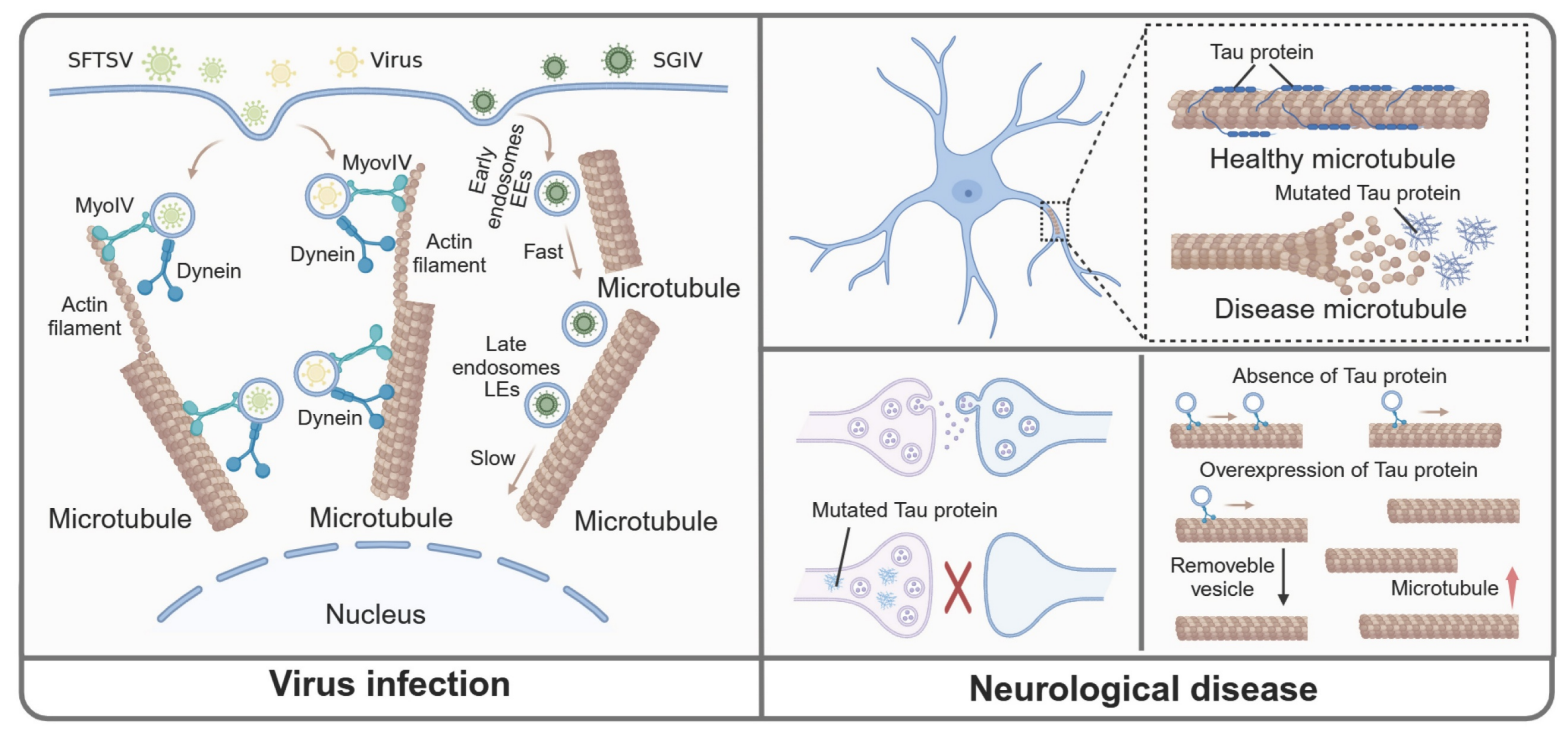
** Microtubule-mediated vesicular transport in viral infection and CNS diseases.** In the virus infection and replication process, SFTSVs are encapsulated within vesicles and are initially transported along actin filaments before transitioning to transport along microtubules. Moreover, the transport of EEs and LEs are increased. The transport of SGIV from the cell membrane to the nuclear area is inhibited during microtubule depolymerization. Normally, Tau combines with microtubule to maintain the normal function of microtubule. In CNS disease, the mutated Tau dissociates from microtubule and relocates to presynaptic, which limit the transport of vesicles. Additionally, the amount of Tau affects the microtubule density and vesicle trafficking. Specifically, the absence of Tau protein decreases microtubule density without altering the total number of mobile vesicles, whereas Tau overexpression increases microtubule density and reduces the overall number of mobile vesicles.

Viruses, characterized by their simple structure, must hijack a host cell to reproduce and survive, a process in which microtubules played a crucial role. During the initial stage of infection, the virus entered the host through endocytosis. Studies showed that several viruses, such as thrombocytopenia syndrome virus (SFTSV) and influenza virus, were encapsulated in vesicles and transported through a "driver switchover" mechanism, which meant these vesicles were initially transported along the actin filaments before being mediated by microtubules 82, 83. Therefore, inhibiting microtubules might be a good choice to halt viral transportation within the host cell. Also, singapore grouper iridovirus (SGIV) was reported to co-located with early endosomes (EEs) and late endosomes (LEs) respectively. Following SGIV infection, the transport of EEs and LEs towards the center of the cell were significantly increased. After applying microtubule depolymerization agent nocodazole, the SGIV transport from the cell membrane to the nuclear area occurring in EEs was significantly inhibited 84. Moreover, during infection with respiratory syncytial virus (RSV), Rab11 regulated intracellular vesicular transport to shuttle viral ribonucleoprotein complexes (vRNPs) from the nucleus to sites of viral assembly and maturation, which was dependent on microtubules. When microtubule depolymerizing agents such as nocodazole were applied, Rab11 was isolated from RNP, thus inhibiting the aforementioned process 20. In summary, we provided additional insights into microtubule-dependent vesicle transport in virus infections, which may inform future research on anti-microtubule drugs targeting these viral infections (**Fig. 4**).

### The role of microtubule-guided vesicular transport in central nervous system (CNS) diseases

Tauopathy is caused by the abnormal accumulation of Tau protein, which is primarily associated with neurodegenerative diseases, including Alzheimer's disease (AD) and Parkinson's disease (PD). The lack of Tau protein could prevent neuronal toxicity and cognitive deficits caused by excitatory toxins in the mouse model of neurodegenerative diseases 85, 86. Furthermore, studies indicated that Tau-targeting therapies might hold promise for treating neurological diseases. A variety of anti-Tau monoclonal antibodies and vaccines have been tested in clinical trials 87. However, as a microtubule-associated protein, Tau could combine with microtubules and regulate the transport of vesicles between presynaptic and postsynaptic. In disease states, the mutated Tau protein dissociated from microtubules and relocated to presynaptic terminals. This alteration caused vesicles to cross-link with presynaptic actin, limiting vesicle trafficking 88. These studies enhanced our understanding of the neurological diseases caused by the Tau protein, and offered valuable insights for future treatment approaches. Moreover, a recent study showed that the amount of Tau protein significantly influenced both microtubule density and vesicle trafficking. In particular, the absence of Tau led to a decrease in microtubule density without altering the total number of motile vesicles. Conversely, the overexpression of Tau increased microtubule density but a decrease in the total number of motile vesicles. Also, it was evident that maintaining Tau protein expression at a certain level exerted a therapeutic effect on neurodegenerative diseases caused by Tau protein 89. Therefore, indiscriminate inhibition of Tau could disrupt normal microtubule-dependent transport of substances within neurons.

In recent years, studies identified that familial mutations in KIF1A, particularly within its conserved motor structure region, potential contributed to HSP 90. This finding warranted further investigation into whether these mutations disrupted the motor mechanism mediated by KIF1A. Contrary to the traditional concept that mutations in the KIF1A ortholog of UNC-104 led to structural defects and subsequently interrupted the anterograde transport of synaptic vesicles. An interesting study has suggested that KIF1A mutations associated with HSP might hyperactivate motor regions to enhance synaptic vesicle anterograde trafficking 91. In addition, the enhancement of anterograde transport mechanism caused seizures phenotype in flies, characterized by increased seizure susceptibility and altered microtubule growth dynamics. Prickle phenotype could inhibit epilepsy, specifically by reducing the expression levels of kinesin responsible for anterograde trafficking vesicle 92.

Microtubule severing enzymes play a crucial role in regulating microtubule growth dynamics under normal physiological conditions. Notably, hereditary spastic paraplegia (HSP) was a neuronal disease caused by gene mutations, such as those in the spastin gene (SPG4) and the kinesin family member 5 (KIF5) gene. These mutations were also associated with other conditions, including spasticity, neurodegeneration, and motor impairment 93, 94. Notably, when spastin was mutated, microtubules underwent elongation with increased tubulin polyglutamylation levels, reducing kinesin binding to microtubules and impairing synaptic vesicle transmission 95. Similarly, disruption of spastin activity reduced the dynamic enrichment of microtubule plus ends and led to misdelivery of synaptic vesicles, thereby impairing microtubule-mediated transport of vesicle cargoes 96. Another member of the microtubule-severing enzyme family, katanin, interacted with CORTICAL MICROTUBULE DISORDERING 4 (CORD4), depending on its p60 subunit, thus promoting the growth and elongation of microtubules to maintain the morphology and function of neurons 97. Under blue light conditions, katanin, cleaved microtubules blocking vesicle transport and even redirecting vesicle trafficking at the cleavage site *in vitro* 98. However, whether katanin interferes with microtubule-mediated vesicle trafficking in neurological diseases remains to be further elucidated. These studies indicated that microtubule proteins, such as Tau and spastin, are critically involved in the vesicle trafficking in CNS diseases (**Fig. 4**).

## Perspectives and conclusions

Since the identification of microtubule-based motor proteins, the study of microtubule-mediated vesicle transport has attracted widespread attention. The directionality and selectivity of microtubule-mediated vesicle transport enable substances to reach their target sites quickly and efficiently. During transport, several factors influence vesicle movement, including microtubule stability, motor proteins selection, and post-translational modifications of microtubules. To date, a growing number of experimental models have been implemented to model microtubule-mediated vesicle transport *in vitro*, enhancing our visualization and understanding of this process. For instance, one study created a kinetic model that measured rate parameters, revealing the reasons behind the slow binding of kinesin-1 to microtubules and identifying the relatively high density of motor proteins required for large-scale vesicle transport. Furthermore, collaboration between different motor proteins can enhance vesicle transport, enabling certain vesicles to achieve long-distance transport by motor protein aggregation in membrane microdomains or through scaffold proteins 99. Although the detailed mechanisms of vesicle transport remain not fully understood, 2D and 3D mathematical models offer a more precise depiction of the process. Emerging technologies may enable precise mapping of microtubule-directed vesicle transport trajectories. For instance, the utilization of high-resolution imaging techniques, such as cryo-electron microscopy and super-resolution microscopy, could reveal interactions among microtubules, motor proteins, and vesicles at higher spatiotemporal resolutions, uncovering their dynamic regulation. Furthermore, integrating computational modeling and artificial intelligence (AI) technologies could advance mathematical frameworks for simulating vesicle transport dynamics and predicting its behavior. These models offer tools to address key questions: (1) how vesicles transfer between microtubules, (2) whether motor or linker protein replacement is involved, and (3) how vesicles bypass obstacles during transport. These insights highlight promising directions for future research.

Vesicle switching between different microtubules requires short-distance transport, an area of research that remains relatively under-explored. Traditionally, the short-distance transport is attributed to the function of microfilaments 6. However, a new study has identified the calcium-sensing dynamic scaffold protein Pclo as a crucial mediator of short-distance transport within the presynaptic membrane, bridging vesicle movement from the reserve zone to the active zone 100. These findings have not only clarified the mechanism of vesicle transport in the presynaptic membrane but also provided a more robust foundation for future research into short-distance vesicle transport. They may also guide research into how a small segment of short-distance transport contributes to the regulation and mediation of microtubule-mediated long-distance transport. In summary, vesicular trafficking is extremely complex, necessitating more comprehensive studies to uncover its underlying mechanisms.

In this review, we have discussed and summarized the abnormal microtubule-mediated vesicle transport in neurons. Building on these findings, targeting microtubule-mediated vesicle transport has emerged as a significant therapeutic approach for treating neurological diseases 101, 102. However, it remains unclear whether these mechanisms exist in other organs and whether they can be targeted for the related diseases. The aforementioned studies indicate that microtubule acetylation acts as an important post-translational modification, which can facilitate vesicle trafficking in neurons. In addition to neurons, epithelial cells, fibroblasts, and immune cells exhibit polarity. Further exploration of the role of vesicle transport in the establishment and maintenance of cell polarity, as well as how its dysfunction leads to cellular disturbances and disease development, holds significant scientific importance. For example, studies could investigate how vesicle transport regulates immune responses (e.g., antigen presentation) and how its dysregulation drives immune disorders--a challenging but promising direction.

Some studies have found that in the process of alcohol-induced liver injury, alterations in tubulin modifications result in increased microtubule acetylation and subsequently destroyed liver function 103. Additionally, CAMSAP2, a protein targeting the negative end of microtubules, mediates liver cancer invasion and metastasis by promoting microtubule acetylation 104. Thus, targeting CAMSAP2-mediated microtubule acetylation may provide a new therapeutic strategy for HCC metastasis. Interestingly, a recent study has identified that microtubule acetylation is also important for the improvement of liver homeostasis and regeneration. Apo-carrying apoptotic vesicles enters the cell through endocytosis, where they form a complex with the hepatocyte Golgi apparatus, promoting microtubule acetylation. Thus, the acetylated microtubules promote hepatocyte cytokinesis, protecting liver homeostasis and regeneration 105. However, given that microtubule acetylation is particularly pronounced in liver diseases, further research is necessary to determine whether and how vesicle transport is also affected in this context. This investigation not only provides new insights into the treatment of liver diseases but also offers a potential therapeutic strategy for other related pathological conditions.

## Conclusions

Microtubules not only facilitate intracellular vesicle transport, including organelles trafficking and apical transport, but also establish direct channels between cells for intercellular communication. Mechanistically, the stability and structural properties of microtubules determine the efficiency of the roads for vesicle trafficking. As motor proteins serve as feet to assist in the vesicle movement along microtubules, the roles of kinesin and dynein in retrograde and anterograde transport, as well as the competitive dynamics, or tug of war, between these motor proteins are concluded. Additionally, diseases linked to abnormal microtubule-mediated vesicle transport are summarized.

## Abbreviations

ATAT1: alpha-tubulin N-acetyltransferase 1
ASOR: asialoorosomucoid
DEX: dexamethasone
ER: endoplasmic reticulum
ERES: ER exit site
Env: envelope glycoprotein
EEs: early endosomes
F-actin: filamentous actin
HTT: Huntingtin
KIF: Kinesin family member
LEs: late endosomes
PICK1: protein interacting with C-kinase 1
PTPN21: protein tyrosine phosphatase N21
PDZK1: PDZ domain containing 1
rOATP1A1: rat OATP1A1
SNARE: the soluble N-ethylmaleimide sensitive factor attachment proteins
HSP: hereditary spastic paraplegia
TNTs: thick tunneling nanotubes
TGN: trans-Golgi network
VEGF: vascular endothelial growth factor
